# Raman Microspectroscopic Analysis of Selenium Bioaccumulation by Green Alga *Chlorella vulgaris*

**DOI:** 10.3390/bios11040115

**Published:** 2021-04-10

**Authors:** Martin Kizovský, Zdeněk Pilát, Mykola Mylenko, Pavel Hrouzek, Jan Kuta, Radim Skoupý, Vladislav Krzyžánek, Kamila Hrubanová, Olga Adamczyk, Jan Ježek, Silvie Bernatová, Tereza Klementová, Alžběta Gjevik, Martin Šiler, Ota Samek, Pavel Zemánek

**Affiliations:** 1 Institute of Scientific Instruments of the Czech Academy of Sciences, v.v.i., Czech Academy of Sciences, Královopolská 147, 612 64 Brno, Czech Republic; pilat@isibrno.cz (Z.P.); ras@isibrno.cz (R.S.); krzyzanek@isibrno.cz (V.K.); hrubanova@isibrno.cz (K.H.); jezek@isibrno.cz (J.J.); berns@isibrno.cz (S.B.); tereza@isibrno.cz (T.K.); bety@isibrno.cz (A.G.); siler@isibrno.cz (M.Š.); osamek@isibrno.cz (O.S.); pavlik@isibrno.cz (P.Z.); 2 Centre Algatech, Institute of Microbiology of the Czech Academy of Sciences, v.v.i., Czech Academy of Sciences, Novohradská 237, 379 81 Třeboň, Czech Republic; mykolamylenko@gmail.com (M.M.); hrouzek@alga.cz (P.H.); 3 Research Centre for Toxic Compounds in the Environment (RECETOX), Faculty of Science, Masaryk University, Kamenice 5, 625 00 Brno, Czech Republic; jan.kuta@recetox.muni.cz; 4 Institute of Physics, Faculty of Physics, Astronomy and Applied Computer Science, Jagiellonian University, Łojasiewicza 11, 30-348 Kraków, Poland; olga.adamczyk@doctoral.uj.edu.pl

**Keywords:** selenium, algae, Raman spectroscopy, EDX, ICP-MS, bioaccumulation, *Chlorella vulgaris*

## Abstract

Selenium (Se) is an element with many commercial applications as well as an essential micronutrient. Dietary Se has antioxidant properties and it is known to play a role in cancer prevention. However, the general population often suffers from Se deficiency. Green algae, such as *Chlorella vulgaris*, cultivated in Se-enriched environment may be used as a food supplement to provide adequate levels of Se. We used Raman microspectroscopy (RS) for fast, reliable, and non-destructive measurement of Se concentration in living algal cells. We employed inductively coupled plasma-mass spectrometry as a reference method to RS and we found a substantial correlation between the Raman signal intensity at 252 cm^−1^ and total Se concentration in the studied cells. We used RS to assess the uptake of Se by living and inactivated algae and demonstrated the necessity of active cellular transport for Se accumulation. Additionally, we observed the intracellular Se being transformed into an insoluble elemental form, which we further supported by the energy-dispersive X-ray spectroscopy imaging.

## 1. Introduction

Selenium (Se) has plenty of commercial and industrial applications, for example, in electronics manufacturing, glass industry, paints, and pigments, etc. Se can be found in three oxidation states—Se(−II), Se(+IV) and Se(+VI) [1]. It is an essential micronutrient for various organisms, including mammals. Se is incorporated in the form of amino acids selenomethionine (SeMet) or selenocysteine (SeCys). SeCys can be present in catalytic sites of essential selenoproteins [2] and plays a key role in the active sites of antioxidant enzymes [3]. These serve for the detoxification and scavenging of reactive oxygen species (ROS), provide anti-neoplastic effect [2], support male fertility, the immune system, and inhibit aging [4].

The difference between the beneficial and toxic concentrations of Se is quite narrow [5]. In adult humans, the daily required dose of Se is about 40 µg, while the toxic dose is approximately 800 μg [6]. However, even though there are some highly Se-contaminated areas, insufficient Se intake is a more common problem among the general population [7]. Se deficiency can lead to cardiomyopathy, cancer, or anemia [8]. Dietary Se can be found, e.g., in seafood, nuts, and meat [6,9]. The amount of Se in the human diet depends on its abundance throughout the lower trophic levels, which reflect the Se concentration in local soils, and is usually low [10]. As a way to mitigate the Se deficiency, pharmaceutical, fitness, and food industries started to introduce products artificially enriched with Se (e.g., crops grown in Se fertilized soil) [6,9]. Yeast and microalgae cultured in Se-enriched media can be easily grown in large amounts under controlled conditions and contain increased proportions of organically bound, highly bioavailable Se [9]. *Chlamydomonas reinhardtii*, *Scenedesmus quadricauda*, multiple *Chlorella* species, and some cyanobacteria were used in Se-metabolism studies and for production of Se-enriched biomass [4,6,11,12,13]. *C. vulgaris* is considered to be one of the most Se-resistant microalgal species [14] which effectively manages to metabolize potentially toxic Se salts into Se-amino acids, lipids, and other low molecular weight compounds [13]. Furthermore, *C. vulgaris* contains a high concentration of proteins, vitamins, and trace elements, which makes it a valuable food supplement [9].

Absorption of selenite (SeO_3_^2−^) or selenate (SeO_4_^2−^) by algal cells is a matter of minutes, while the incorporation of Se in different organic forms occurs within days [10]. Transportation of Se to the cells is provided by two major routes; the phosphate transport mechanism is used by SeO_3_^2−^, whereas SeO_4_^2−^ utilizes the sulfate transporters and channels. However, in an environment with high sulfate or phosphate concentrations, the selectivity for Se decreases, and the nominal ligands are preferred by the transport systems [15]. When cultivated in a Se-rich medium, algal cells can neutralize the harmful effect of Se by converting it to less toxic volatile methylated compounds—dimethylselenide and dimethyldiselenide [12], but only a small part of the absorbed Se is transformed into these compounds [16]. Alternatively, Se can be intracellularly accumulated in a reduced form as elemental Se^0^ [2], which forms multiple allotropes with a monoclinic ring or polymeric chainlike molecules [17]. Thanks to the ability of algae to accumulate Se, some species could be potentially used for phytoremediation of Se-contaminated areas [14].

Multiple tools and techniques were developed to control Se content in food and to monitor the environmental pollution [18]. There is a considerable amount of published research specifically focusing on the analysis of different Se compounds and their concentration in various organisms. Detection of Se in microbes was accomplished in the 1970s, when Gerrard et al. studied Se accumulation in *E. coli* via electron microscopy [19]. Other studies focused, e.g., on Se-rich yeasts with inductively coupled plasma–mass spectrometry (ICP-MS) for Se detection [20] and plants, such as Indian mustard [21] or garlic [22] for Se speciation. These two studies utilized high-performance liquid chromatography (HPLC) coupled with ICP-MS and in tandem with electrospray mass spectroscopy. One of the common methods for analysis of total selenium in a biomass, is ICP-MS after digestion by nitric acid and hydrogen peroxide [11,23,24]. However, Se cannot be speciated this way since it is oxidized (mainly to selenate). HPLC-ICP-MS or gas chromatography (GC)-ICP-MS are used for speciation after acidic hydrolysis with methansulfonic acid, or enzymatic hydrolysis of the biomass [25,26].

The analytical techniques described above are very reliable and sensitive with low limits of detection (LOD) and quantification (LOQ), but the sample preparation and operation procedures are relatively expensive and time-consuming. Therefore, it is very important to develop a non-invasive means for faster monitoring of Se content in microalgae with minimal sample handling procedures, ideally in vivo. In our previous studies, we were able to detect different biomolecules (such as carotenoids or lipids) in living algal cells, in real time, by Raman microspectroscopy (RS) [27,28]. RS is a versatile tool for determination of chemical composition of various materials, chemicals, or biological samples [29]. RS exploits the inelastic Raman scattering of an incident laser light to record the vibrations of molecular bonds in the sample. It can be performed in living cells with no interference from water and minimal sample preparation [29,30]. The focus of this work is to explore the capacity of RS to quantify Se in algal samples. To our best knowledge, there is no other publication dealing with RS based detection of Se in any form in algal cells up to this date. The resulting methodology may be used for fast and simple food supplements or environmental analysis. The results obtained from RS measurements were validated by comparison to data from ICP-MS and energy-dispersive X-ray spectroscopy (EDX).

## 2. Materials and Methods

### 2.1. Cultivation of Se-Enriched C. vulgaris

Green freshwater microalga C. vulgaris G-120 (registered as C. vulgaris BEIJ., 1996/H 14, CCALA 30,001, Culture Collection of Autotrophic Organisms, Institute of Botany, Třeboň, Czech Republic) was cultivated heterotrophically for 4 days in conical flasks (300 mL starting volume). The flasks were placed on an orbital shaker (120 rpm) and grown in the dark at 25 °C in a batch mode. The initial biomass density was about 2 g dry weight (DW) per liter. The cultivation medium was described by Mylenko et al. [6].

Growth of the cultures was monitored by optical density at 750 nm, which is directly proportional to the biomass DW according to the empiric formula: DW [g/L] = OD_750_/2. Based on the optical density measurements, Se was supplemented to the culture twice a day in the form of Na_2_SeO_3_ (Alfa Aesar, Karlsruhe, Germany) in concentrations of 0.5, 1, 4, 8, and 16 mg Se/g DW. The pH of the medium was 7.5.

### 2.2. Analysis of Se Accumulation Mechanism in Living and Dead Cells

We examined if the ability of *C. vulgaris* to bioaccumulate Se ions from the medium depends on an active transport across the cell membrane. Two cultures of *C. vulgaris* were cultivated separately according to the protocol above. One culture was subsequently inactivated by heating to 60 °C for 30 min (named “Dead”), while the second culture was left untreated (named “Living”). Death of the cells was confirmed by fluorescein diacetate (Invitrogen, Thermo Fisher Scientific, Waltham, MA, USA) vital staining (modified from Serra-Maia et al.) [31]. The cultures were cultivated for 4 days and sampled on days 1, 2, and 4. Half of the samples were controls with no added Se. The other half was cultivated with an addition of 4 mg Se/g DW twice a day. The biomass was washed twice with the Se-free cultivation medium (without glucose) to eliminate the extracellular Se and prepare for the analyses. The washing procedure ensured that we observed only the Se accumulated inside the cells.

### 2.3. Sample Collection and Preparation for ICP-MS and RS

10–50 mL of the biomass suspension was collected in plastic tubes, centrifuged, and lyophilized for ICP-MS [6] or frozen in liquid nitrogen for RS and kept in the freezer at −80 °C.

### 2.4. Inductively Coupled Plasma Mass Spectrometry (ICP-MS) Analysis

The total content of selenium in algal biomass was determined by ICP-MS (Agilent Technologies Japan Ltd, Tokyo, Japan). Prior to the analysis, the samples were digested with nitric acid and hydrogen peroxide in a microwave digestion system (MWS 3+ Berghof, Eningen, Germany). The accuracy of the method was verified by analysis of lyophilized selenium-enriched yeast certified reference material (SELM-1 CRM) obtained from National Research Council Canada (Ottawa, ON, Canada), and the measured value 1.96 ± 0.13 mg/g was found within the range of the certified value 2.059 ± 0.064 mg/g. The precision of the method (relative standard deviation) was estimated by replicate analysis of SELM-1 CRM and several algal biomass samples and was typically found in single units of percent [6].

### 2.5. Sample Handling Device

**Figure 1:**
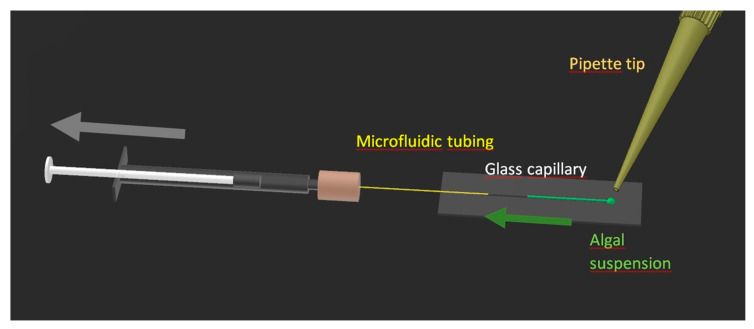
A capillary microfluidic device for observation and exchange of algal samples under stable optical settings of the Raman measurement. The microfluidic tubing was approximately 30 cm long to allow easy manipulation and simultaneously avoid unintentional movement of the slide while operating the syringe. Image parts not to scale.

The intensity of the Raman signal strongly depended on the position of the laser focus within the sample volume. We constructed a device to allow undisturbed Raman measurements on a series of samples without the need to move the microscope table or refocus during the sample exchange, see Figure 1. A glass capillary with a square cross section and inner diameter of 0.5 mm (length: 50 mm, outer diameter: 0.7 mm) (VitroCom, NJ, USA) was attached to a microscope slide by UV-curing adhesive (Loctite AA 3494, Henkel Adhesive Technologies, Düsseldorf, Germany). Microfluidic PEEK tubing, outer diameter 0.36 mm (1572, IDEX Health & Science, Oak Harbor, WA, USA), was inserted into the capillary on one side and secured with the same adhesive. The opposite end of the tubing was connected by a Luer adaptor (P-662, IDEX Health & Science, Oak Harbor, WA, USA) to a 1 mL syringe (Omnifix-F solo, B. Braun, Melsungen, Germany), which was used to load the algal sample into the capillary and to flush it with deionized sterile water. The opposite end of the capillary served for the loading of the algal sample.

### 2.6. Raman Microspectroscopy

The algal samples were left to thaw spontaneously at room temperature. The sample handling device was put into the Raman microspectrometer (InVia Reflex, Renishaw, Wotton-under-Edge, UK) and 20 μL of the undiluted algal suspension was dropped near the open end of the square capillary and carefully aspirated to about 1/3 of its length by the attached syringe. The microscope was then focused into the sample in the capillary, 20 μm below the glass-medium interface. We used a microscope objective with 20× magnification, NA 0.40 (N PLAN EPI, Olympus, Tokyo, Japan). Raman spectra of the samples were measured with 30 s integration time at 785 nm excitation wavelength and 70 mW of the laser power in the sample plane. Multiple cells were measured simultaneously, providing an averaged spectrum. We used in-house data processing software based on MATLAB (MathWorks, Natick, MA, USA). We used iterative polynomial fit (order: 10, passes: 10) for fluorescence removal and Savitzky-Golay algorithm (order: 2, frame length: 7) to remove spectral noise. We defined the Raman signal of Se as the peak area A between 250–255 cm^−1^. We normalized the spectra on the peak area A at 470–480 cm^−1^. The Raman signal at 470–480 cm^−1^ was directly attributed to SiO_2_ from the glass capillary, see Figure S1. Its intensity was indirectly proportional to the distance from the glass surface, see Figure S2. The normalization therefore effectively compensated for the minor differences in the focus position. Visualization of spectra processing can be seen in Figure S3. All RS measurements of Se concentration are presented as a relative Raman intensity I_RR_ (Se), which was defined as the ratio between Se (A_250–255_) and SiO_2_ (A_470–480_) peak areas. The number of measured spectra for each variant ranged from 40 (days 1 and 2) to 60 (day 3).

### 2.7. Scanning Electron Microscopy (SEM) and EDX Analyses

The algal samples were air-dried and covered in a sputter coater Q150T (Quorum Technologies, Laughton, UK) by a 6 nm layer of chromium and imaged in the SEM Magellan 400 L (FEI, Thermo Fisher Scientific, Waltham, MA, USA) using Octane Elect Super EDX detector (AMETEK, Berwin, IL, USA). The beam energy was set to 5 keV, probe current 3.2 nA, and magnification 5000x was used for EDX maps capture. The distance of the EDX detector from the sample was 40 mm and the sample was facing the detector by the stage tilt of 30°.

EDX is a method allowing elemental analysis of the sample. X-rays excite electrons in the inner electron shells, creating an electron hole in the process. The hole is then refilled with an electron from the outer shell, releasing X-rays specific for this energy difference, which is given by the unique structure of each element. That allows measurements of elemental composition of a sample. When combined with SEM, EDX can provide high resolution maps of distribution of individual elements in the sample. Quantification of Se from EDX maps was performed with image analysis software ImageJ (National Institute of Health, Bethesda, MD, USA) [32]. The original pictures were converted to 8-bit gray scale, and then to a binary mode by thresholding. An EDX map of a sample with no added Se was used for the threshold value determination. The threshold was found to be 30 (out of 255 levels), reducing the brightness of every image by 12%, which eliminated the background noise, resulting in a representation of the Se clusters as spots spread on a homogeneous background. The proportion of these spots relative to the whole image area was plotted against the Se concentrations obtained from ICP-MS.

## 3. Results and Discussion

We analyzed the algal cells of *C. vulgaris* with RS to assess its suitability as a complementary method to ICP-MS for Se quantification in algal samples.

### 3.1. RS Quantification of Se in Algal Cells and Validation by ICP-MS

**Figure 2:**
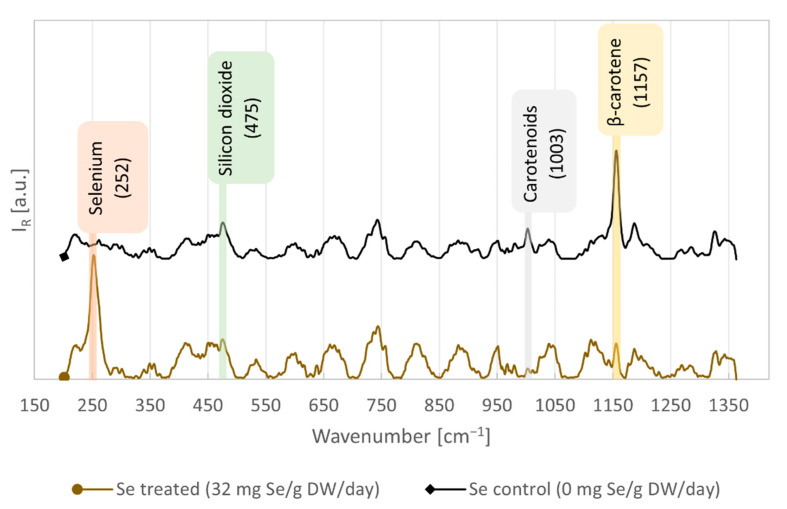
Raman spectra of algal samples cultivated with maximal Se concentration (32 mg Se/g DW/day) or no (0 mg Se/g DW/day) added Se. The signal of Se-Se vibrations was observed at 252 cm^−1^. Each spectrum was averaged from 20 measurements. See Supplementary Materials Figures S5 and S6 for more Raman spectra from the algal samples.

When RS was performed on samples with high Se concentration, we observed the sample illuminated with the laser to change its color to a reddish tone. It indicated that the laser radiation has induced the photoreduction processes leading to the production of the red allotrope of elemental Se^0^ from SeO_4_^2−^ and SeO_3_^2−^, or from its organic forms. We compared the Raman spectra from the algal cells treated with a high concentration of Se to those of the control cells with no added Se, see Figure 2. The algae with high Se concentration exhibited intensive Raman signal of Se at 252 cm^−1^. According to Jadhav et al. [33] and Goldan et al. [34], this peak represents the ring vibration of the monoclinic Se_8_ molecule. A peak of β-carotene at 1157 cm^−1^ was reduced in intensity compared to the control cells, also see Figure S4 (Supplementary Materials). A possible explanation is that abundant Se in the form of SeO_3_^2−^ blocked membrane transporters for phosphates and thus inhibited production of various phosphates and pyrophosphates (PP), such as isopentenyl PP [35], that are necessary for β-carotene synthesis. Therefore, β-carotene could potentially serve as an indirect indicator of Se presence in the sample. The assignments of the selected dominant spectral features are presented in Table 1. We found that the Raman peak at 252 cm^−1^ is the most reliable indicator of Se concentration in our algal samples after comparing it to the results from ICP-MS. The peaks with no assignment in Table 1, none of which were used for the subsequent analyses, may be influenced to a different extent by the residual etalon ripple effect, a frequent artefact of signals acquired over long integration time, see also Figures S5 and S6 (Supplementary Materials).

Our observation of elemental Se in the cells corresponds with a finding that when the cells are not able to further produce Se-containing amino acids from SeO_3_^2−^ they start to metabolize SeCys to form Se^0^ using enzyme SeCys lyase [2,15], or oxidize Se^2−^ (produced by sulfite reductase) before it is incorporated into SeMet and SeCys. Different transformation of SeO_3_^2−^ to elemental Se^0^ was described in sonicated pea chloroplasts. The mechanism revolves around SeO_3_^2−^ reductive assimilation by an illuminated chloroplast (in our case illuminated by laser) and unlike the previous pathway, it does not involve sulfite reductase. Instead, the electrons are generated during the photolysis of water together with O_2_. These electrons are transported through NADP(H) (nicotinamide adenine dinucleotide phosphate) and GSH (glutathione)/GSSG (glutathione disulfide), to reach SeO_3_^2−^ as their final acceptors, forming Se^2−^. Afterwards, Se^2−^ can be transformed into SeCys in the presence of O-Acetylserine, or be non-enzymatically oxidized to Se^0^ [36]. Our inability to detect organic forms of Se could also be due to the laser induced photodegradation of selenoproteins, resulting in formation of individual SeCys molecules, which could be then reduced to Se^0^ by the lyase enzyme. This hypothesis is supported by the observed effect of light intensity and oxygen concentration on the proteolysis of an isolated oat chloroplast [37].

**Figure 3:**
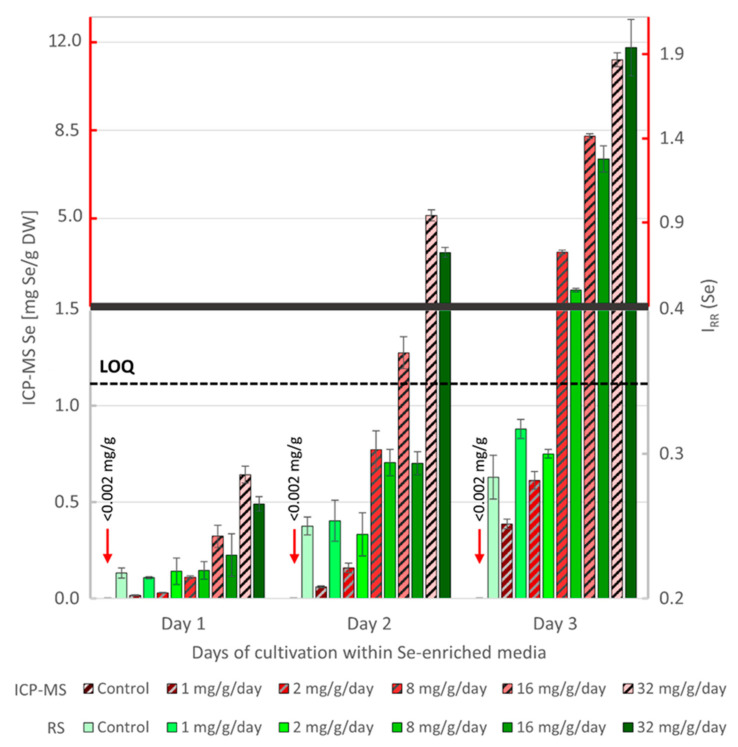
Comparison of Se concentrations in algal samples during the cultivation measured by ICP-MS (red/grey stripes) and RS (green). Each bar represents the mean value for specific added Se concentration (3 values for ICP-MS; 120–160 spectra for RS). The thick horizontal grey line indicates the change of scale of both Y-axes for better clarity of low Se concentration measurements. The scale for higher Se concentrations is red for further visual separation. Horizontal dashed line represents the LOQ. Error-bars: 2 standard deviations (SD).

We compared the intensity of the Raman signal of Se I_RR_ (Se) from the algal cells to the total Se content, determined by ICP-MS, from the algal biomass sampled during the cultivation experiment, see Figure 3. The Se-exposed cells progressively accumulated Se during the experiment. We detected Se levels in the range between 0 to 12 mg Se/g DW using ICP-MS. The RS signal linearly correlated with the ICP-MS data in Se concentrations above 1 mg Se/g DW. Using the equation LOQ = 10 * (SD/Sp) [42], where SD is standard deviation of the response of the whole curve and Sp is its slope, we established the LOQ to be 1.12 mg Se/g DW. We were able to detect Se by RS, even below the LOQ, but the unexplained variability in the measurements of low Se concentrations prevented us from a reliable quantitative interpretation.

**Figure 4:**
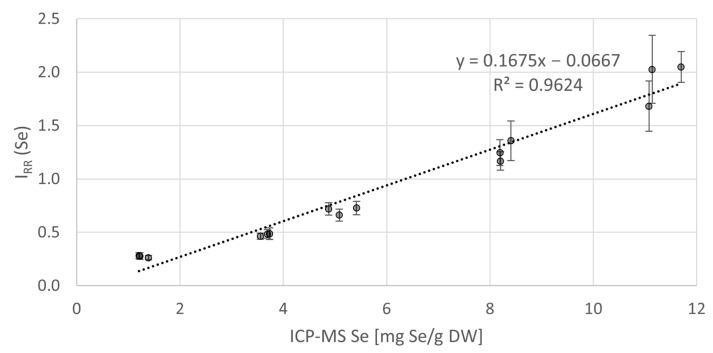
Relation between the Se concentrations measured by ICP-MS and the corresponding Raman signal intensity I_RR_ (Se) values. Each data-point represents the mean value from all the processed Raman spectra (40–60) obtained from a single sample. Error-bars: 2 SD.

We calibrated the Raman signal of Se on the ICP-MS reference while excluding the data-points below the LOQ, see Figure 4. We fitted the data with a linear regression curve, resulting in a regression coefficient value R^2^ = 0.96. In samples with less than 2 mg Se/g DW, near the LOQ calculated according to the above formula, the linear regression curve substantively deviates from the data. The rest of the data-points showed relative errors in the range of 1–16% (average: 9%). The high R^2^ value suggests that RS could be used to quantify Se in algal cells above the determined LOQ in the experimental setup presented in this study. However, precise Se quantification based on a linear calibration is possible, roughly, from 3 mg Se/g DW. Deviations from the trend are mainly due to the imperfect background removal caused by the variable fluorescence intensity of each sample.

### 3.2. Comparative RS Analysis of Se Uptake Dynamics by Living and Dead Algal Cells

**Figure 5:**
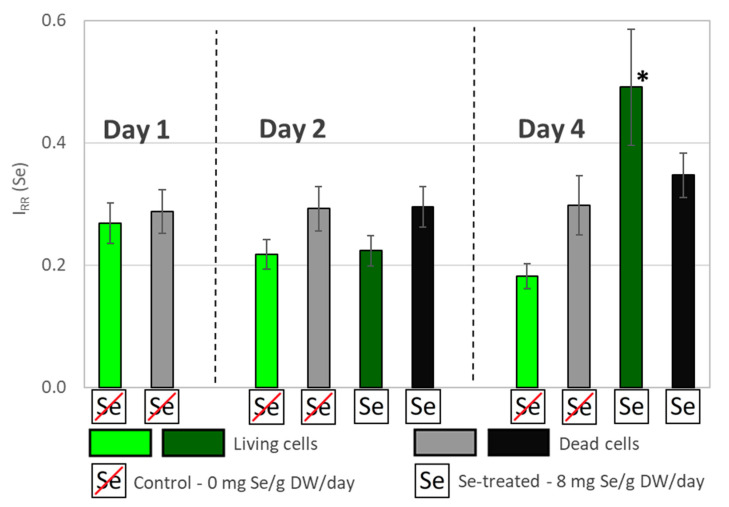
Se accumulation by regular (living) and heat inactivated (dead) algal cells. Only the living (dark green) and dead (black) cells on day 4 accumulated significant amounts of Se during the cultivation period. The asterisk represents the sample that was significantly (*p* = 0.05) different from the control when analyzed with a paired *t*-test. Error-bars: 2 SD.

To further test the effectiveness of the Raman-based Se quantification in algae, we measured the accumulation of Se in living and dead cells during a four-day Se treatment. The dynamics of Se accumulation in the living and dead cells are presented in Figure 5. The significance of the results was evaluated by a paired T-test with a *p*-value 0.05. The only I_RR_ (Se) values above the LOQ were obtained on day 4 for living and dead cells treated with Se. Since the control values were below the LOQ, only a qualitative interpretation of these results was possible. Apparently, the accumulation of Se was significant only in the living cells. The increased Se signal intensity was observed only on day 4. This may indicate that Se was poorly absorbed by the cells until it reached a certain threshold concentration. Dead cells on day 4 show a small change in I_RR_ (Se), which could be due to a passive diffusion of Se into the cell.

### 3.3. Verification of the Elemental Se Deposition in Algal Cells by SEM and EDX Analysis

**Figure 6:**
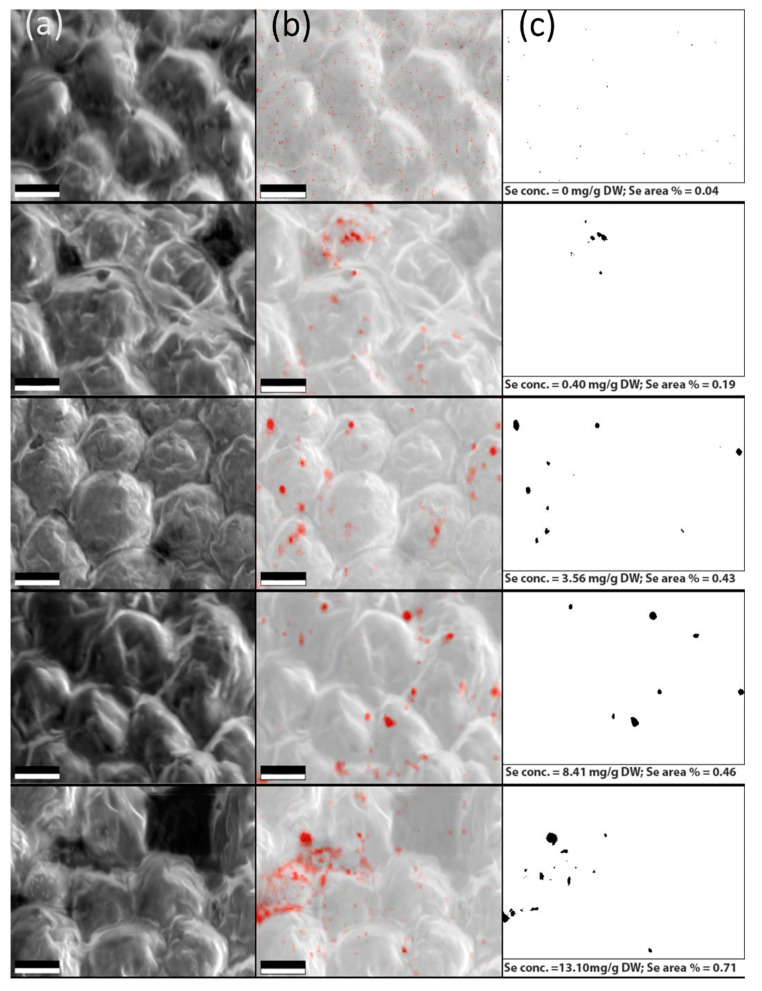
(**a**) Se supplemented *C. vulgaris* cells imaged by SEM. (**b**) EDX scan of the identical areas with Se signal presented in red—the saturation of these spots is directly proportional to Se concentration. (**c**) The binary version of the EDX scan after a stringent noise removal. The black spots represent the Se signal above the threshold. The Se concentrations detected by ICP-MS and the percentages of area coverage by the Se signal are listed below the images. Removal of the background noise revealed only the biggest Se clusters. The samples were selected to cover the entire interval of Se concentrations used in our experiments. Scale bar: 2 μm.

**Figure 7:**
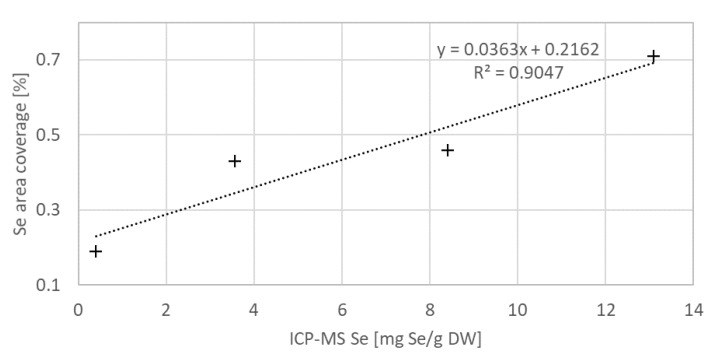
Se concentrations in the selected algal samples derived from the EDX map (on Figure 6), expressed in per cent of the image area covered by the Se signal, in relation to the Se respective concentrations measured by ICP-MS. Quasilinear dependence was found between the two data-sets.

To examine the Se distribution in *C. vulgaris*, SEM and EDX imaging was performed on a selection of the samples. Figure 6 presents the electron microscope images of the algal cells (a), the same areas mapped in EDX spectroscopic mode with highlighted Se occurrence (b), and the same area in binary mode after thresholding of the EDX map (c). The samples contained increasing Se concentrations from top to bottom—see the subscript under each figure in the (c) column. The EDX signal of Se inside the algal cells appears clustered, which suggests that even at 0.4 mg/g DW, the Se ions were too concentrated to be processed completely into soluble organic forms, causing some of the Se to, instead, be reduced into its elemental form and stored in patches around the cell. The clusters may be composed of Se nanoparticles [43]. We also examined whether the total area of the Se signal clusters in the binary pictures reflects the actual Se concentration in the sample measured by ICP-MS, see Figure 7. To assess this relation, we used a linear regression. We found the regression coefficient value was R^2^ = 0.90. While this represents a definite trend, its linearity could not be determined properly because of the non-uniform distribution of Se combined with the limited amount of obtained EDX maps.

## 4. Conclusions

We successfully employed Raman microspectroscopy for the quantification of Se in living cells of *C. vulgaris* intended for human food supplementation. We found direct linear proportionality between the total Se concentration measured by ICP-MS and the Raman signal at 252 cm^−1^ assigned to Se-Se bond vibrations in Se_8_ monoclinic rings, with an experimentally determined regression coefficient of 0.96. The LOQ was established as 1.12 mg Se/g DW. We also observed the Raman signals of carotenoids, such as β-carotene at 1157 cm^−1^, to decrease in response to elevated Se concentration. We used the Raman-based approach to measure the difference in Se absorption between living and dead algal biomass. Our measurements reaffirmed the necessity of active transport for Se accumulation in cells. During Raman microspectroscopy of the algal biomass, we visually observed the laser-induced formation of red elemental Se. When we visualized the distribution of Se in the algal cells with combination of SEM and EDX mapping, we observed a tendency of elemental Se to form dense, randomly scattered clusters. The number and total area of the observed clusters were directly proportional to Se concentration in the cultivation medium. This reflects the limited effectiveness of biological conversion of inorganic Se sources into the organic forms of Se, especially at high Se concentrations in the medium. The fluorescence of the algal sample was one of the main factors that prevented us from reaching LOQ under 1 mg Se/g DW. The measurement capabilities of the employed approach could be improved using a low-fluorescence fused quartz capillary, since the glass capillary contributed to the total fluorescence of the sample by up to about 10% in the area of interest (200–1100 cm^−1^), compare Figure S7 to Figure S3 (Supplementary Materials). Further improvements could be possibly reached with prolonged sample bleaching or a more sophisticated background removal algorithm. However, each method has its own pitfalls.

The established methods of Se quantification in biomass, such as ICP-MS, usually offer the possibility of speciation, superior sensitivity, and excellent reproducibility, but they require laborious, destructive, time-consuming, and expensive sample preparation. In contrast, we show that Raman microspectroscopy presents an attractive capability to provide fast, non-invasive, on-line monitoring of accumulation of total Se by microalgae. This includes the production cultures used for food supplements or species present in Se contaminated water bodies.

## Figures and Tables

**Table 1:** Assignments of selected Raman peaks in the spectra of *C. vulgaris* supplemented with Se.

Peak Wavenumber [cm^−1^]	Assignment	Reference
252	**Selenium** Se-Se stretching in monoclinic Se_8_ rings	[33]
475	**silicon dioxide** Si-O-Si rocking and bending	[38]
1003	**Carotenoids** CH_3_ in-plane rocking	[39,40]
1157	**β-carotene** C-C stretching, C-H in-plane bending	[39,40,41]

## Data Availability

The authors confirm that the data supporting the findings of this study are available within the article.

## Supplementary Materials

The following are available online at https://www.mdpi.com/article/10.3390/bios11040115/s1, Figures S1–S7.
