# Supplementary material for: Raman Microspectroscopic Analysis of Selenium Bioaccumulation by Green Alga Chlorella vulgaris
Source: Biosensors (Basel). 2021 Apr 10;11(4):115. doi: 10.3390/bios11040115 (PMC8069876; doi:10.3390/bios11040115)
Supplement: Supplementary file 1 [file biosensors-11-00115-s001.zip › biosensors-1166466-supplementary.docx]

**Supplementary Materials**

**
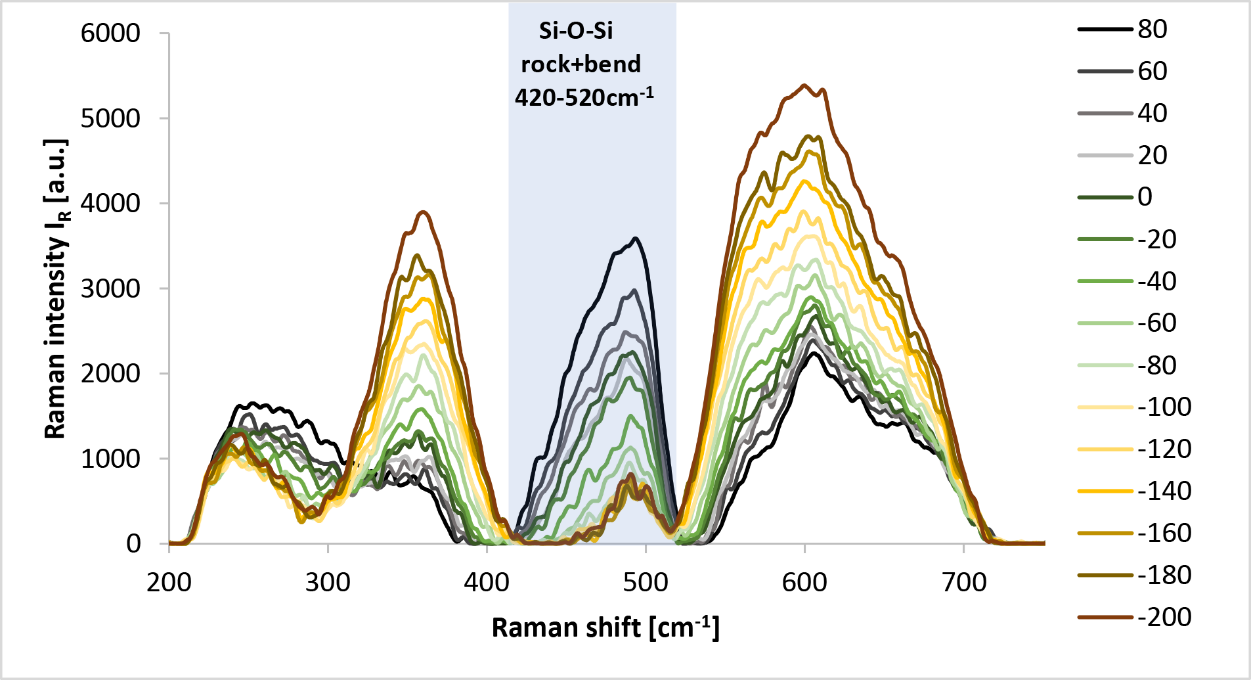
**

**Figure S1.** Raman spectra of a water-filled glass capillary used for Raman measurements of the algal samples. The spectra were recorded from different depths (in micrometers) as indicated in the legend. Zero represents the boundary between the sample and the glass wall, with more negative numbers towards the center of the capillary. The area of Si-O-Si bond vibrations was highlighted around 475 cm^-1^.


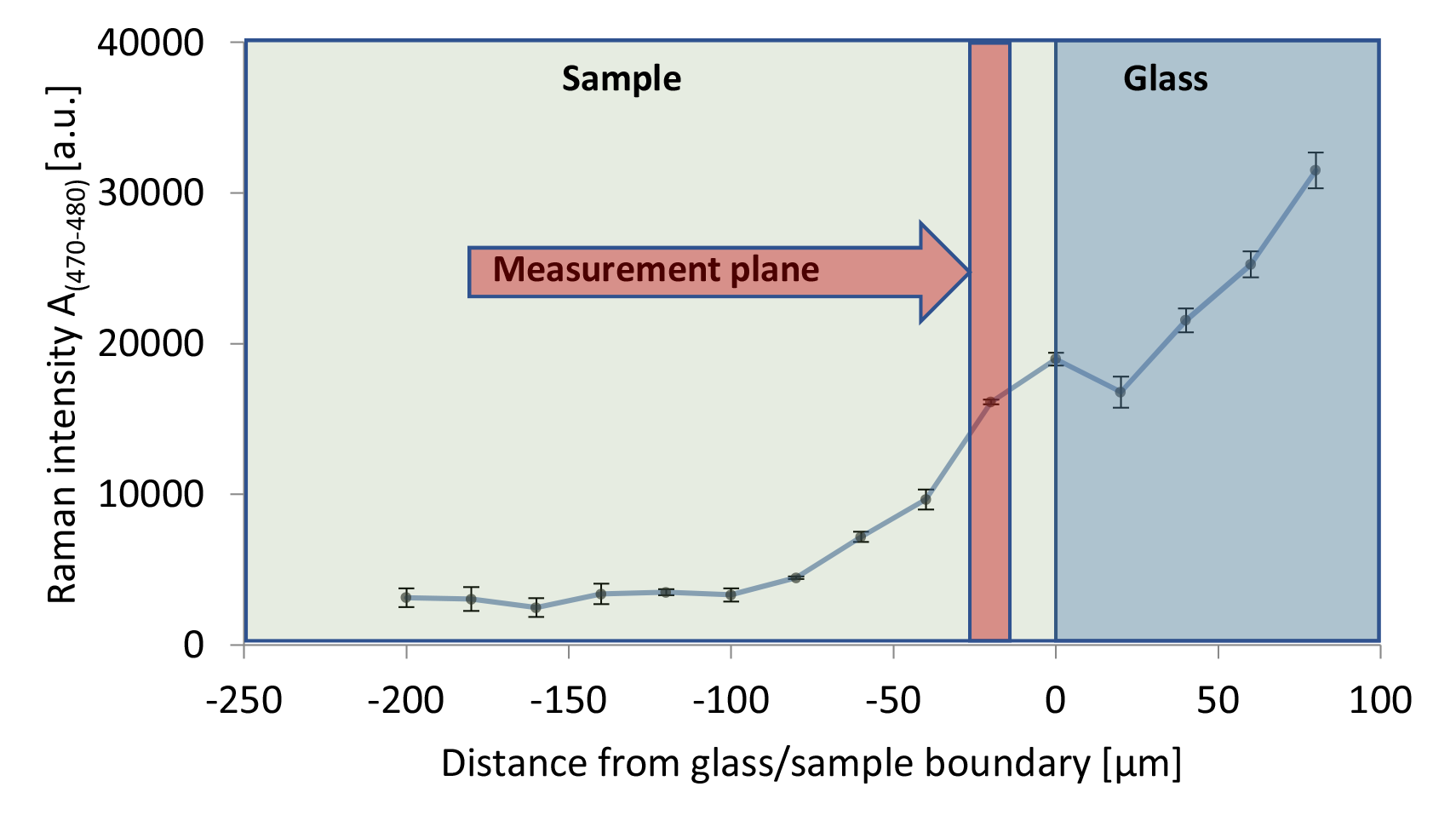


**Figure S2**. The intensity profile of the Raman signal of SiO_2_ vibration at 475 cm^-1^ from the sample-carrying glass capillary. The y-axis represents the Raman signal intensity integrated over the spectral area 470-480 cm^-1^. The x-axis represents the distance from the glass-sample boundary. The location of the focal plane for measurement of Raman spectra from algal samples is shown as a red stripe about 20 μm below the glass/sample boundary.

**Figure S3**. Processing of Raman spectra from *Chlorella* cells (the original spectrum is an average of 180 spectra). All spectra were processed the same way. Normalization was made to the peak area ranged between 470-480 cm^-1^.


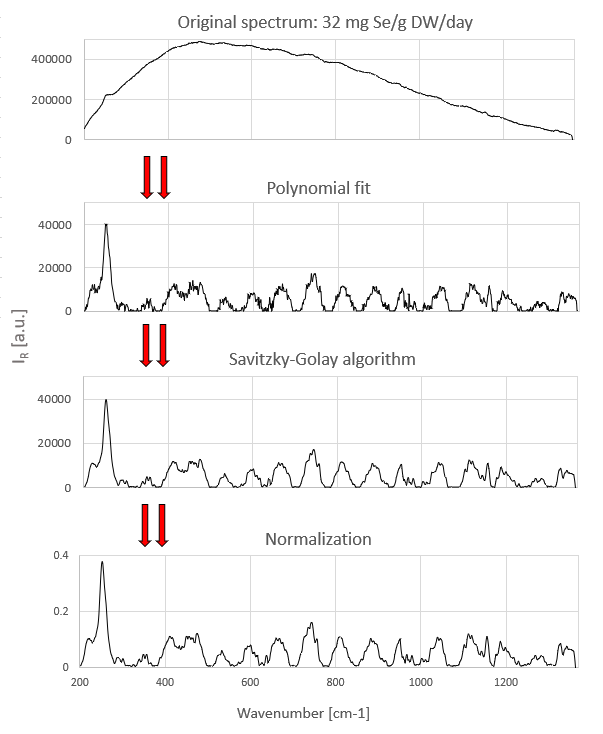

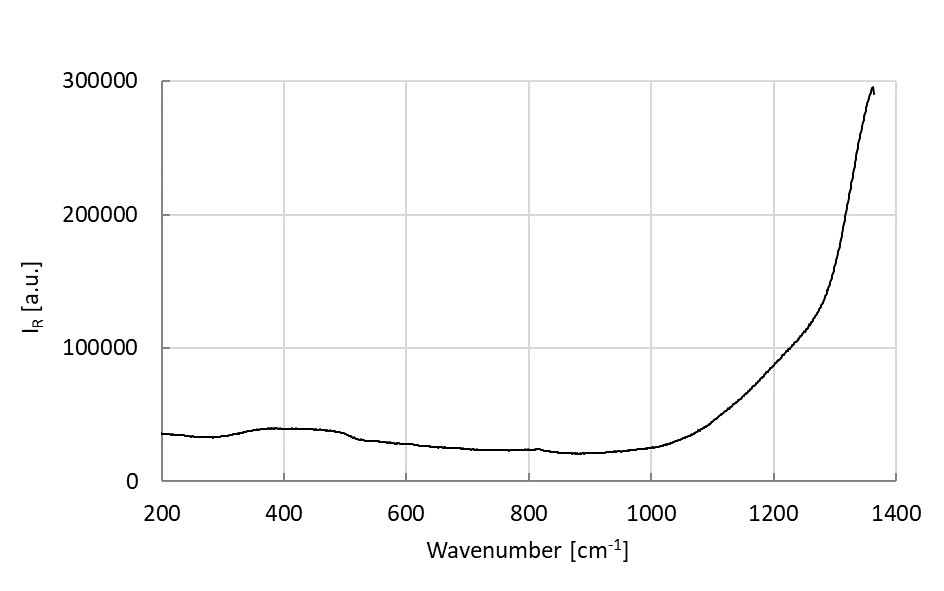


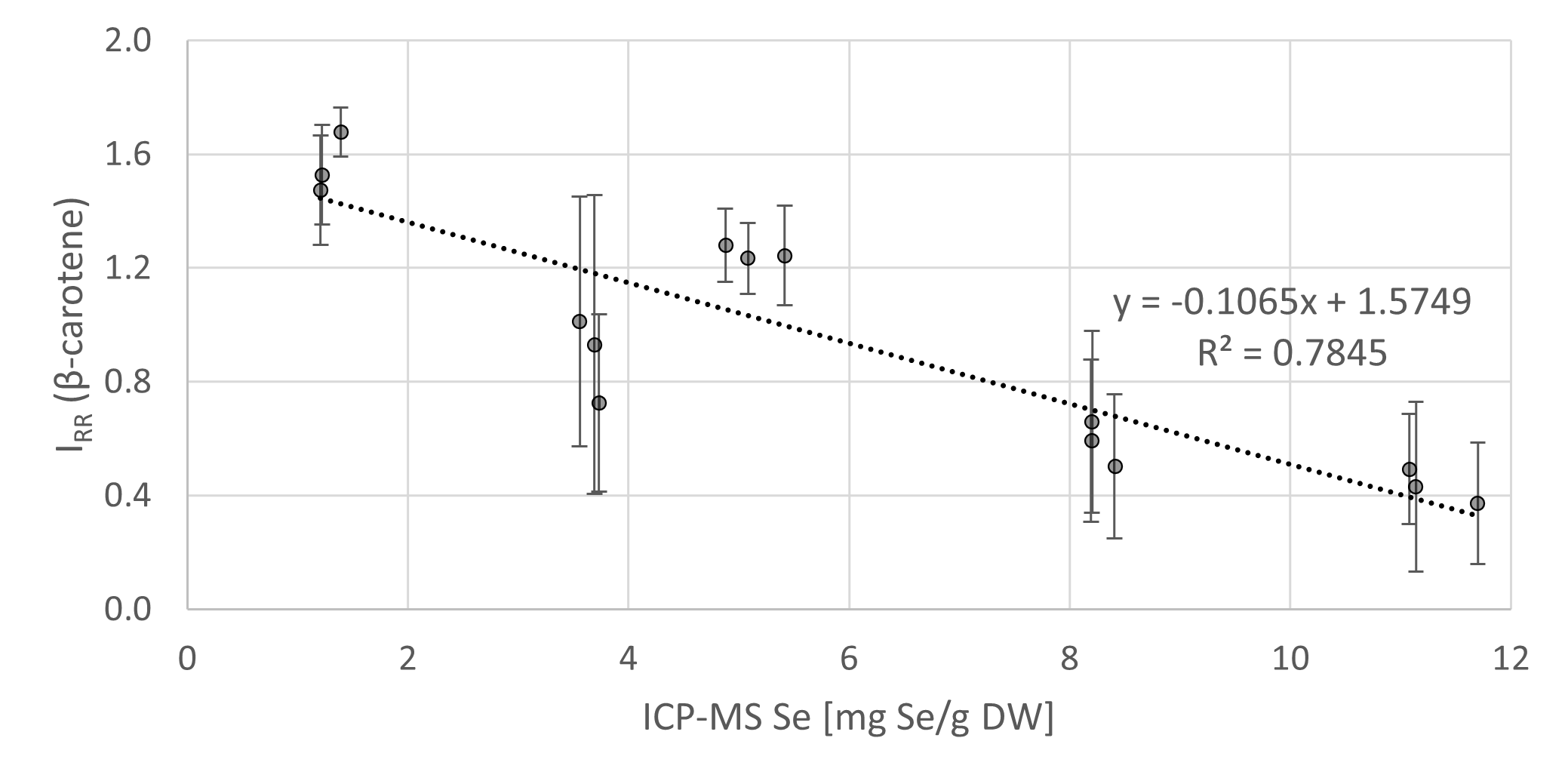
**Figure S4**. Relation between the Se concentrations in *Chlorella* measured by ICP-MS and the corresponding intensity of the Raman signal I_RR_ of β-carotene. Each data-point represents a mean value from all the processed Raman spectra (40-60) obtained from a single algal sample. Error-bars: 2 SD


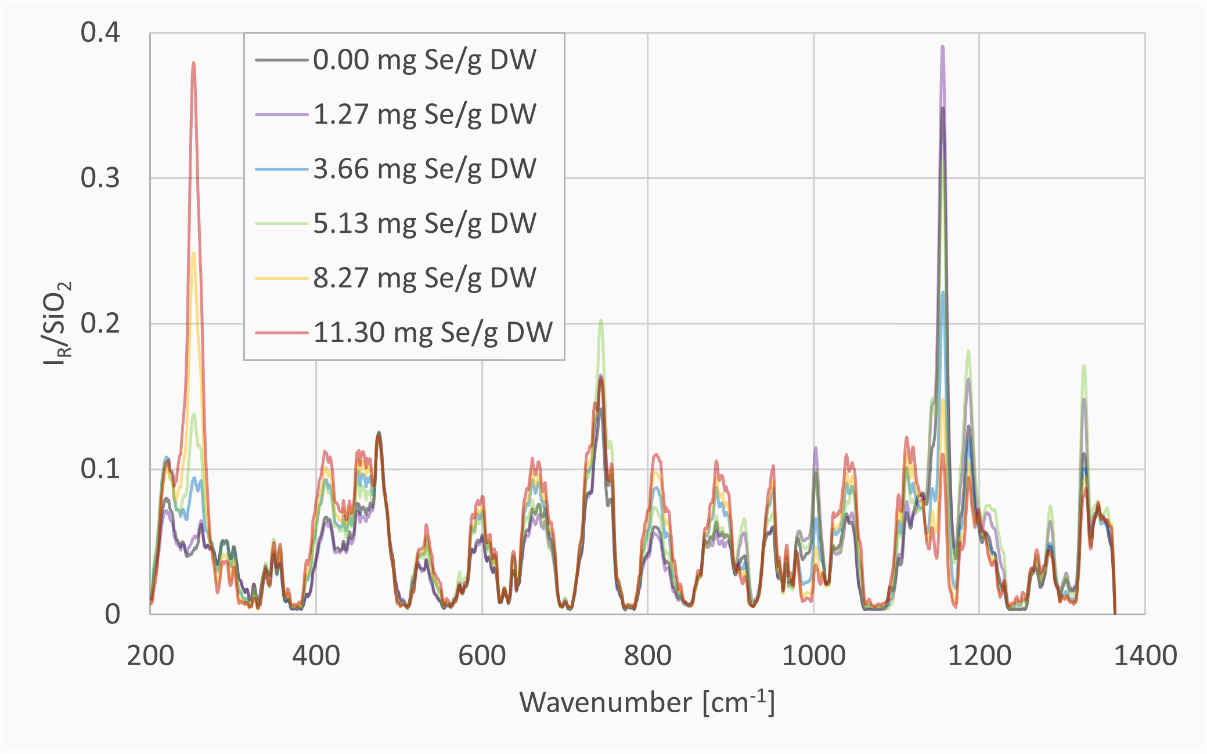


**Figure S5**. Average Raman spectra of *Chlorella* cells. The peak of Se at 252 cm^-1^ is proportional to the concentration of Se in the cells. The peak at 1157 cm^-1^ is indirectly proportional to Se concentration in the algal sample. 180 spectra were averaged per each depicted spectrum. The values of Se concentration were averaged from three ICP-MS measurements each. Raman spectra were normalized at a peak area 470-480 cm^-1^.


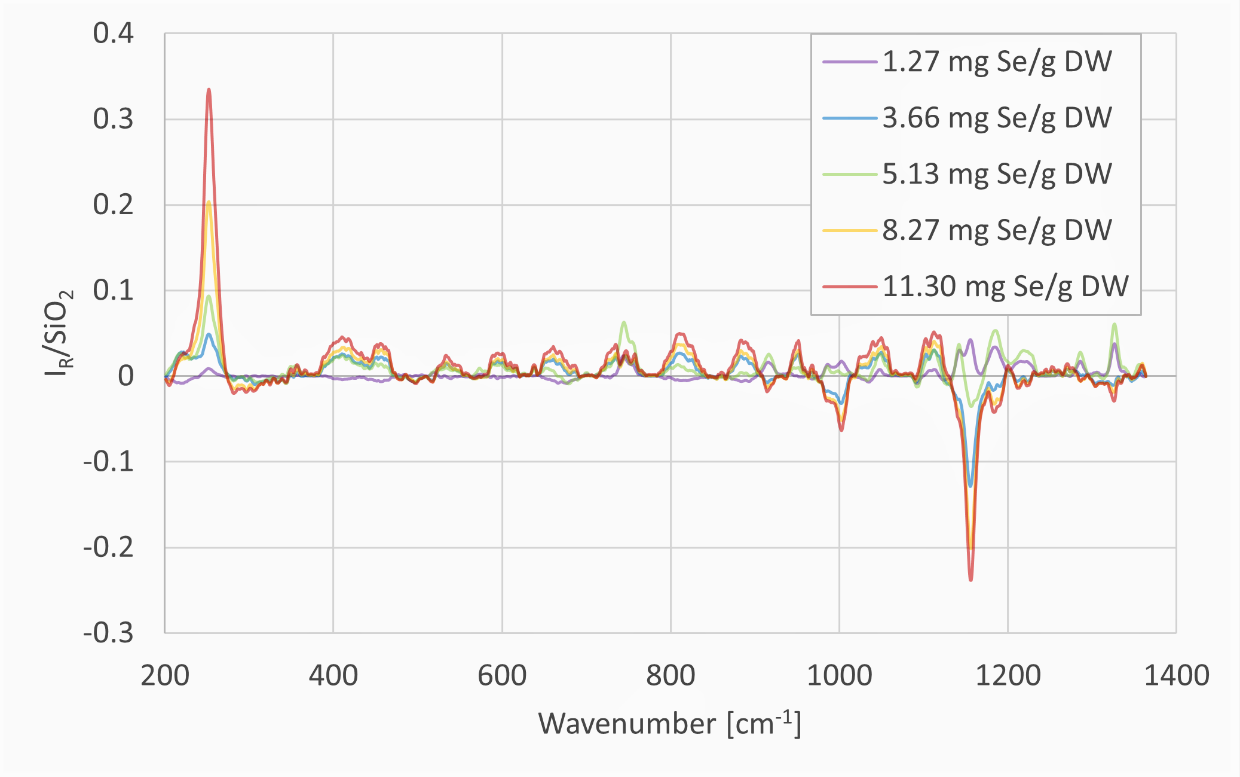
**Figure S6**. Average differential Raman spectra of samples with Se levels above the LOQ. An average Raman spectrum from *Chlorella* cells with no added Se was subtracted from each average spectrum from cells with added Se. The peak of Se at 252 cm^-1^ is proportional to the concentration of Se in the sample. The peak at 1157 cm^-1^ is indirectly proportional to Se concentration in the algal sample. 180 spectra were averaged per each depicted spectrum. The values of Se concentration were averaged from three ICP-MS measurements each. Raman spectra were normalized at a peak area 470-480 cm^-1^.


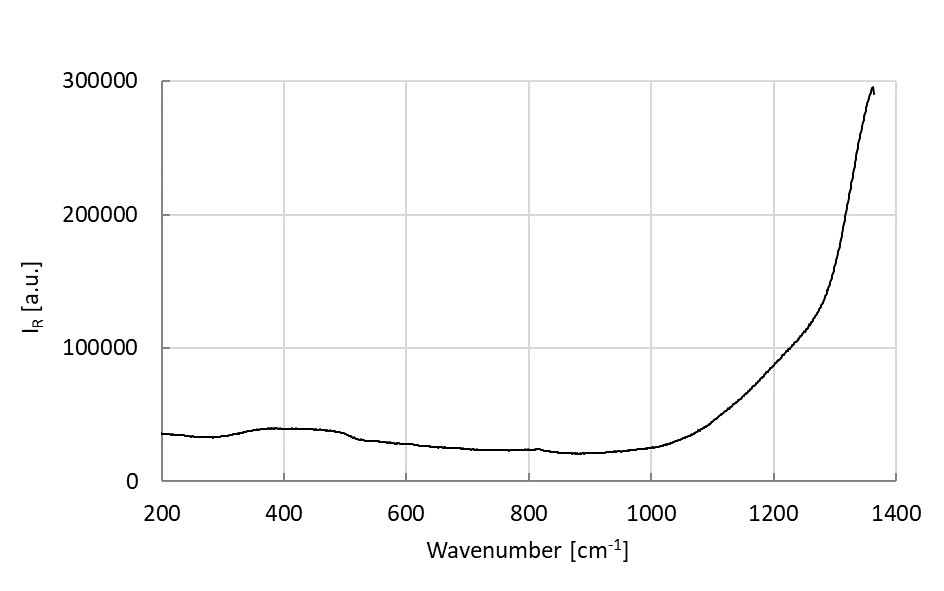


**Figure S7**. Unprocessed Raman signal (containing fluorescence), acquired from the glass capillary filled with deionized water. The focus of the microscope objective was positioned 20 μm under the water-glass interface. All the instrumental settings were identical with the measurement of the algal samples.
